# The impulse response of optic flow-sensitive descending neurons to roll m-sequences

**DOI:** 10.1242/jeb.242833

**Published:** 2021-12-06

**Authors:** Richard Leibbrandt, Sarah Nicholas, Karin Nordström

**Affiliations:** 1 Neuroscience, Flinders Health and Medical Research Institute, Flinders University, GPO Box 2100, 5001 Adelaide, SA, Australia; 2 Department of Neuroscience, Uppsala University, Box 593, 751 24 Uppsala, Sweden

**Keywords:** Motion detection, Lobula plate tangential cells, *Eristalis tenax*

## Abstract

When animals move through the world, their own movements generate widefield optic flow across their eyes. In insects, such widefield motion is encoded by optic lobe neurons. These lobula plate tangential cells (LPTCs) synapse with optic flow-sensitive descending neurons, which in turn project to areas that control neck, wing and leg movements. As the descending neurons play a role in sensorimotor transformation, it is important to understand their spatio-temporal response properties. Recent work shows that a relatively fast and efficient way to quantify such response properties is to use m-sequences or other white noise techniques. Therefore, here we used m-sequences to quantify the impulse responses of optic flow-sensitive descending neurons in male *Eristalis tenax* hoverflies. We focused on roll impulse responses as hoverflies perform exquisite head roll stabilizing reflexes, and the descending neurons respond particularly well to roll. We found that the roll impulse responses were fast, peaking after 16.5–18.0 ms. This is similar to the impulse response time to peak (18.3 ms) to widefield horizontal motion recorded in hoverfly LPTCs. We found that the roll impulse response amplitude scaled with the size of the stimulus impulse, and that its shape could be affected by the addition of constant velocity roll or lift. For example, the roll impulse response became faster and stronger with the addition of excitatory stimuli, and vice versa. We also found that the roll impulse response had a long return to baseline, which was significantly and substantially reduced by the addition of either roll or lift.

## Abstract (precis)

**Summary:** The impulse response of hoverfly optic flow-sensitive descending neurons to roll m-sequences reaches its time to peak within 20 ms and slowly returns to baseline over the next 100 ms.

## INTRODUCTION

When animals move through the world, their own movements generate widefield optic flow across their eyes. Such optic flow cues are useful for staying on a straight trajectory, for returning to a home, or when performing other navigational tasks. The processing of self-generated optic flow has been especially well investigated in insects. Indeed, one of the most commonly applied models for motion detection was based on the steering response to widefield motion, known as the optomotor response, in a beetle (Hassenstein and Reichardt, 1956).

In flies, optic flow is processed by 45–60 lobula plate tangential cells (LPTCs; e.g. Pierantoni, 1976), which have been studied since at least the 1960s (e.g. Bishop and Keehn, 1967). The best described of these belong to the horizontal system (HS) and vertical system (VS; e.g. Hausen, 1982; Hengstenberg et al., 1982). Many other insects, including hawkmoths (Stöckl et al., 2016), bumblebees (Mertes et al., 2014) and bees (Paulk et al., 2014), also have optic flow-sensitive neurons in their optic lobes that are physiologically and anatomically similar to the fly neurons. *Drosophila* have 6 VS cells (Scott et al., 2002), blowflies have 10 (Hengstenberg et al., 1982) and hoverflies probably have a similar number (Buschbeck and Strausfeld, 1997). Anatomical work shows that VS cells synapse with DNDC 3–5, as well as DNDC 1–4 (also referred to as DNOVS4; Gronenberg and Strausfeld, 1990). Later work, using receptive field mapping, physiological response properties and dye coupling, showed that VS cells synapse with the DNOVS2 neuron (Suver et al., 2016; Wertz et al., 2008, 2009b), which is also called DNp22 (Namiki et al., 2018). DNOVS2 projects to areas in the thoracic ganglion that control neck, wing and leg movements (Namiki et al., 2018). Analysis of how the sensory information that passes through the descending neurons is transformed to motor control is a rapidly expanding field (Ache et al., 2019a; Cande et al., 2018; Chen et al., 2018), with population code control of behavior being likely (Ache et al., 2019b; Gonzalez-Bellido et al., 2013; Levi and Camhi, 2000).

In blowflies, the pre-synaptic input of DNOVS2 mainly comes from VS5 and VS6 (Wertz et al., 2009b), whereas it comes from VS2 and VS3 in *Drosophila* (Suver et al., 2016). In addition, the blowfly DNOVS2 receives contralateral input from V2, which can only be measured when DNOVS2 is depolarized (Wertz et al., 2009b), and from the ocelli (Gronenberg et al., 1995; Haag et al., 2007). As expected from this input (Karmeier et al., 2006), and because of the non-linear receptive field component from V2 (Wertz et al., 2009a,b), blowfly DNOVS2 neurons respond strongly to roll, but also to lift. In hoverflies, the presumed DNOVS2 counterpart is referred to as the type 2 optic flow-sensitive descending neuron (Nicholas et al., 2020). The hoverfly type 2 neuron, like DNOVS2, responds strongly to roll, but also to other types of optic flow, such as lift (Nicholas et al., 2020; Suver et al., 2016). DNOVS2 and the hoverfly type 2 neuron respond to visual motion in a direction-selective manner, similar to the pre-synaptic VS cells (Nicholas et al., 2020; Suver et al., 2016; Wertz et al., 2009a,b), but appear to be tuned to higher velocities. LPTCs adapt strongly to continuous motion and show a direction-selective after-effect (Kurtz et al., 2009; Nordström et al., 2011). In contrast, the hoverfly type 2 neuron does not adapt as strongly to continuous motion and, in addition, it shows strong persistent firing following preferred-direction stimulation (Nicholas and Nordström, 2020).

One way to study optic flow sensitivity is to use white noise techniques (Ringach and Shapley, 2004; Roy et al., 2015). White-noise stimuli are powerful in that they comparatively rapidly provide the data needed to extract the spatio-temporal response dynamics of a neuron or a visual behavior. Maximal length shift register sequences (m-sequences), for example, consist of a series of integers, with each integer being either −1 or +1. These specify the stimulus impulse polarity, which in the case of motion vision could be two opposite directions of motion (Aptekar et al., 2014). An m-sequence follows very strict rules. If it is of the order *n* it has a length of 2*^n^*−1, and has the following characteristics (Ringach and Shapley, 2004): (1) there are 2*^n^*^−1^ occurrences of +1 and 2*^n^*^−1^−1 occurrences of −1; (2) every possible subsequence of +1s and −1s of length *n* occurs only once; (3) the product of an m-sequence and a time-shifted copy of itself is the same m-sequence, but time shifted.

Optic flow responses to white noise have been described from several different fly species and neurons. For example, the blowfly H1 neuron's spike-triggered average to yaw, roll and pitch has a time to peak (TTP) of ca. 20 ms, and a slow return to baseline after about 100 ms (Roy et al., 2015). In addition, the yaw spike-triggered average is decreased in amplitude by the addition of either roll or pitch (Roy et al., 2015). The *Drosophila* HS cell impulse response to sinusoidal gratings controlled by an m-sequence also peaks early, and then decays back to baseline in less than 400 ms, following an exponential decay with a time constant of 65 ms (Schnell et al., 2014). The impulse response of hoverfly HS cells peaks after 18 ms, and decays back to baseline over the next 100 ms (Lee et al., 2015). These studies thus suggest that white noise techniques, including m-sequences, are a powerful method for quantifying the spatio-temporal response dynamics of optic flow-sensitive neurons, such as LPTCs. However, the impulse responses of optic flow-sensitive descending neurons have not been described. Considering that descending neurons show some interesting differences compared with their presynaptic LPTCs (Kurtz et al., 2009; Nicholas and Nordström, 2020; Nordström et al., 2011), this warrants further investigation.

To address this, we recorded extracellularly from male *Eristalis tenax* type 2 optic flow-sensitive descending neurons. These neurons respond particularly well to roll optic flow (Nicholas et al., 2020). Hoverflies are extremely good at performing stabilizing head movements in response to body roll perturbations, with important input from the visual system (Goulard et al., 2015). If the type 2 optic flow-sensitive descending neuron is the hoverfly homolog of DNOVS2, as suggested (Nicholas et al., 2020), it is likely to project to the part of the thoracic ganglion that could control such head movements (Suver et al., 2016). It is thus not unreasonable to assume that the type 2 neuron could be involved in these rapid head roll-stabilizing reflexes. To understand more about the spatio-temporal dynamics of these neurons, we thus extracted the impulse response to m-sequences controlling roll motion. As the H1 yaw spike-triggered average has been shown to be affected by the addition of other types of optic flow (Roy et al., 2015), we additionally quantified the roll impulse response after adding constant roll or lift motion, and found that the roll impulse response was strongly affected by this.

## MATERIALS AND METHODS

### Animals and electrophysiology

We recorded from 13 male *Eristalis tenax* (Linnaeus 1758) hoverflies, 0.5–10 months old, reared and housed as described earlier (Nicholas et al., 2018). At the start of the experiment, the animal was immobilized ventral side up with a beeswax and resin mixture, and a small hole cut at the anterior end of the thorax. A sharp polyimide-insulated tungsten electrode (2 MΩ, Microprobes, Gaithersburg, MD, USA) was inserted into the cervical connective, with mechanical support given by a small wire hook. The animal was grounded via a silver wire inserted into the ventral cavity, which also served as the recording reference.

We recorded from type 2 optic flow-sensitive descending neurons, which were identified by their receptive field and physiological response properties (Nicholas et al., 2020). Extracellular signals were amplified at 1000× gain and filtered through a 10–3000 Hz bandwidth filter on a DAM50 differential amplifier (World Precision Instruments), with 50 Hz noise removed with a HumBug (Quest Scientific, North Vancouver, BC, Canada). The data were digitized via a Powerlab 4/30 (ADInstruments, Sydney, NSW, Australia) and acquired at 40 kHz with LabChart 7 Pro software (ADInstruments).

### Visual stimuli

Visual stimuli were created with custom software, written in Matlab (MathWorks 2017) and making use of the Psychophysics toolbox (Brainard, 1997; Pelli, 1997). The software was executed on a Dell Alienware computer running Ubuntu 16.0.4. Stimuli were displayed on a linearized Asus LCD screen (Asus, Taipei, Taiwan) with a spatial resolution of 2560×1440 pixels, running at a frame rate of 165 Hz. The fly was positioned upside-down in front of the screen, with the long axis of its body perpendicular to the screen, and its head at a distance of 6.5 cm from the screen, resulting in a final resolution of 155 deg azimuth×138 deg elevation.

**Fig. 1.**
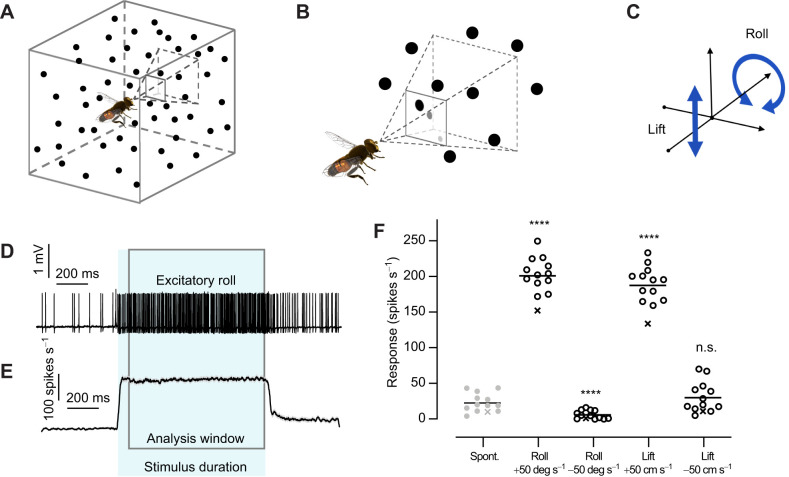
**A starfield stimulus that simulates optic flow.** (A) We simulated a cube with 4 m sides, with the hoverfly placed in the center. The space was filled with randomly placed spheres with a diameter of 2 cm, at a density of 100/m^3^. Note that the figure is not to scale. (B) The spheres simulated to be anterior of the hoverfly were projected onto the flat visual stimulus display, with circle size and brightness used to indicate distance in the virtual space. Note that the figure is not to scale. (C) We used the space to simulate upwards and downwards lift, and roll in the clockwise or counter-clockwise direction. (D) Raw data trace from a type 2 optic flow-sensitive descending neuron in response to roll motion at 50 deg s^−1^. (E) The response across 13 type 2 neurons to roll motion at 50 deg s^−1^. The spike histogram shows the mean±s.e.m. response at 1 ms resolution after smoothing with a 20 ms square-wave filter. In D and E, the shading shows the peri-stimulus duration and the boxed area is the analysis window. (F) Type 2 neurons are significantly excited by roll in the preferred direction and by downwards lift, and significantly inhibited by roll in the opposite direction (one-way ANOVA, followed by Dunnett's multiple comparison test, *N*=13, ****P<0.0001). The crosses show the data from a neuron that was excluded from further analysis because of its lower spike rate.

The visual stimulus was a moving starfield of grayscale circles against a white background. The stimulus simulated a three-dimensional cloud of spheres of diameter 2 cm (Nicholas et al., 2020), positioned on each trial at random locations within a cube with 4 m sides around the hoverfly, at an average density of 100 spheres m^−3^ (Fig. 1A, not to scale). The spheres anterior of the hoverfly were projected onto the two-dimensional screen representation to produce each successive frame of the stimulus, resulting in a collection of circles displayed in each frame (Fig. 1B, not to scale). The circle diameter was inversely related to the straight-line distance from the hoverfly, so that spheres that were located closer were rendered as larger circles. Circle brightness was inversely linearly interpolated between black (6 cm distance) and white (2 m distance), so that closer spheres were rendered as darker circles. Spheres at a linear distance smaller than 6 cm from the hoverfly were not rendered on screen.

To simulate optic flow, all spheres in the starfield were moved in unison between each frame of the simulation. For roll, spheres were rotated around an axis along the hoverfly body and perpendicular to the screen. For lift, spheres were translated up or down along the vertical axis (Fig. 1C). The starfield stimulus moved either continuously for 1 s or as controlled by an m-sequence. Between each stimulus presentation, the screen remained blank for a minimum 3 s.

During m-sequence stimulation, the starfield moved in a series of incremental clockwise or counter-clockwise roll stimulus impulses, at the refresh rate of the screen (165 Hz). Each m-sequence was of the 8th order, thus having a total length of 2^8^−1 (255) stimulus impulses. As m-sequences are circular (Aptekar et al., 2014), and in order to minimize the effect of onset response transients, the m-sequence was extended to a length of 400, but only the final 255 stimulus impulses were used in the data analysis. The direction of each stimulus impulse was determined according to the randomly selected m-sequence: clockwise or counter-clockwise depending on whether the corresponding value in the m-sequence was positive or negative. Each stimulus impulse rotated the starfield by 0.33 deg, referred to as ‘Roll 33’, unless otherwise indicated. When displayed at 165 Hz, this would give a velocity of 50 deg s^−1^ if all stimulus impulses moved in the same direction, a velocity that drives optic flow-sensitive descending neurons strongly (Nicholas et al., 2020). For each hoverfly, Roll 33 m-sequences were repeated over 8 successive trials. Every trial, across hoverflies and conditions, used a unique randomly generated m-sequence. One exception was made for the purpose of validating our analysis: the second Roll 33 m-sequence was always identical.

In some conditions, the Roll 33 m-sequence was combined with a constant velocity (Theobald et al., 2010b) of lift or roll. For roll, we used a constant velocity of 25 or 50 deg s^−1^. When run at the 165 Hz refresh rate of our monitor, this corresponded to a series of 0.15 or 0.3 deg stimulus rotations. For lift, we used a constant velocity of 50 cm s^−1^. When run at 165 Hz, this corresponded to a series of 0.3 cm stimulus translations.

All stimuli were recorded with the stimulus software as well as with a photodiode placed on the screen. If any stimulus frames were dropped during presentation of a trial, that entire trial was discarded from further analysis.

### Data analysis

Raw voltage data were spike sorted and converted to a spike train with LabChart 7 Pro software (ADInstruments), using spike amplitude and width.

For starfield stimuli moving continuously (Fig. 1D–F), we quantified the mean spike rate for the entire stimulus duration, after removing the first 100 ms of the response to avoid any initial onset transients (Nicholas et al., 2020). The spontaneous rate was calculated for 0.5 s immediately preceding stimulus onset.

For each m-sequence trial, the impulse response of the neuron was calculated under the assumption that the response *y*(*t*) is related to the stimulus *x*(*t*) by convolution with a linear response kernel *h*(*t*) representing the impulse response, i.e.:

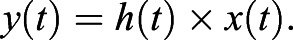


The impulse response was therefore calculated as the cross-correlation of the stimulus m-sequence with the neural response (see Reid et al., 1997), represented by the binary-valued spike train. For each condition in each hoverfly, the mean impulse response was then calculated as the mean over all trials of the condition. As the spike train is a noisy estimate of the instantaneous firing rate, the calculated cross-correlation exhibited noise. To obtain the eventual linear kernel, the mean correlation was smoothed using a Gaussian filter with a 5 ms window.

**Fig. 2.**
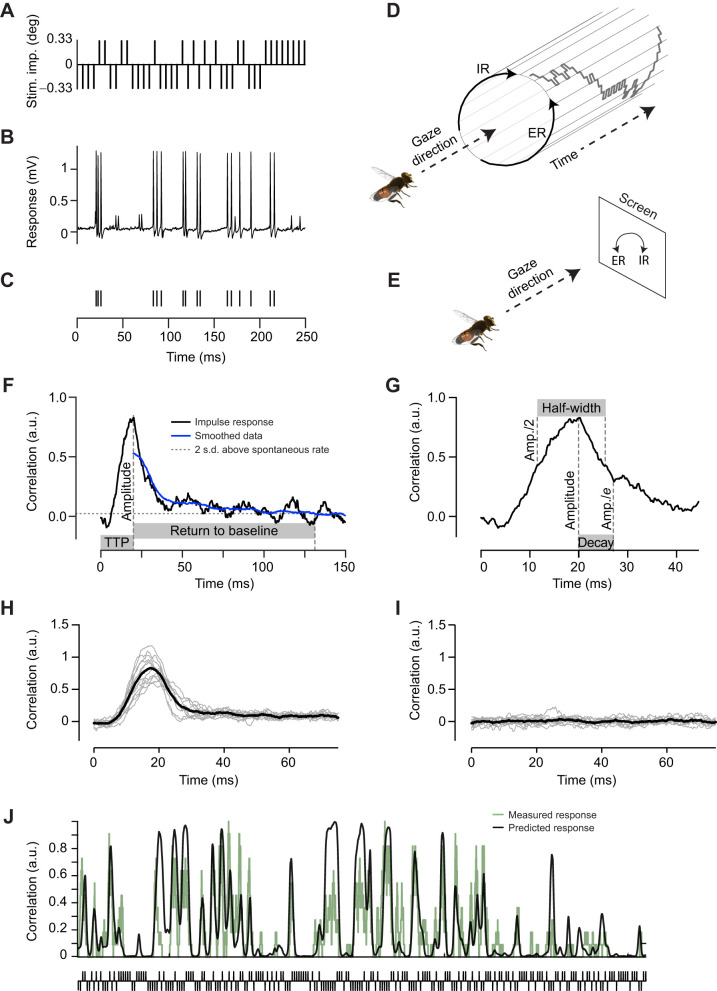
**An m-sequence can be used to quickly and robustly extract the impulse response to roll optic flow.** (A) To control roll motion, we used m-sequences which described a series of 0.33 deg stimulus impulses in the preferred (positive) and anti-preferred (negative) direction. The pictogram shows an extract of an m-sequence. (B) The raw response of a single type 2 neuron to the m-sequence extract shown in A. (C) The resulting spike train after spike sorting the data in B. (D) From the hoverfly's point of view, the m-sequence controls incremental rotations in the excitatory (ER) and inhibitory (IR) direction over time (note, rotation increments are not to scale). (E) Excitatory roll (ER) rotates in the counter-clockwise direction on the screen, whereas inhibitory roll (IR) rotates in the clockwise direction. (F) The roll impulse response extracted from one neuron, with the amplitude and time to peak (TTP) highlighted, as well as the return to baseline method (blue). (G) The definitions of half-width and decay. (H) The roll impulse response extracted from 12 individual neurons (gray) and the mean (black). (I) The roll impulse response extracted by using shuffled spike trains from 12 individual neurons (gray), as well as the resulting mean (black). (J) We validated the m-sequence method by using the average impulse response calculated from each individual neuron separately, to predict the response to a shared m-sequence seen by all neurons, and correlated this prediction with the measured response across all other neurons. Shown is the predicted response for one neuron (black), and the recorded mean response over all other neurons (green). The correlation was in this case 0.719. a.u., arbitrary units.

From each neuron's mean impulse response in each condition, we extracted several parameters (Fig. 2F,G). ‘Amplitude’ was calculated as the maximum value of the impulse response filter, and ‘TTP’ as the time interval from stimulus presentation to the time at which maximum amplitude was attained. To calculate ‘return to baseline’, we first used a Gaussian filter with a broad time window (25 ms) to smooth the stimulus–response correlation (Fig. 2F, blue). Return to baseline was calculated as the time interval from the TTP to the point where the smoothed correlation first returned to within two standard deviations of its baseline mean (Fig. 2F, dotted line). ‘Half-width’ was defined as the width of the impulse response at 50% maximum amplitude (Fig. 2G). ‘Decay’ was defined as the time interval from the TTP to the point when the amplitude decreased to 1/*e* where *e* is the natural number, also referred to as the exponential constant (Fig. 2G). We also quantified the average ‘spike rate’ during the 255 stimulus impulses of the m-sequence.

### Validation of impulse response calculation

For validation, we did two things. First, we used an identical Roll 33 m-sequence that was presented to every hoverfly, which was not used for determining its impulse response. We used this impulse response, from each hoverfly, to predict the response to the common m-sequence of the second trial. To account for the non-linearity in the response prediction, the input m-sequence was convolved with the impulse response to obtain a linear ‘generator signal’ following the method in Chichilnisky (2001). Subsequently, we estimated the non-linearity function by binning the generator signal values into discrete bins and calculating the mean firing rate inside each bin. Predictions were generated by convolving the shared m-sequence of the second trial with the impulse response, then passing the output into the non-linearity function to yield a predicted spike rate.

We then calculated the Pearson correlation of the prediction with the average response across all other hoverflies. We did this by summing all spike trains together, and then smoothing them by using a sliding mean over a 200 ms window. This process was repeated, each time using data from a different hoverfly, until all hoverflies had been used, and the median correlation was calculated.

Second, we quantified the Roll 33 impulse response from shuffled spike trains. We did this by randomizing the timing of all spikes recorded in response to each Roll 33 m-sequence (i.e. if *K* spikes occurred during the presentation of the m-sequence, we selected *K* random time points within the total duration of the m-sequence), before extracting the impulse response as above. In addition, the ‘shuffled impulse response’ was used for prediction by convolving it with the shared m-sequence of the second trial, then passing the output into the non-linearity function to yield a predicted spike rate, which was again correlated with the mean response across all other hoverflies. This process was repeated 20 times for each neuron, and the mean of the 20 correlation values was calculated.

### Inclusion criteria and statistical analysis

We recorded from 13 type 2 descending neurons in 13 male hoverflies. If a neuron's mean firing rate in response to preferred direction roll was below 80% of the reported mean response (199±15 spikes s^−1^, mean±s.e.m.; see Nicholas et al., 2020), i.e. below 159 spikes s^−1^, all data from that hoverfly were discarded from further analysis. This criterion led to data from one hoverfly being discarded (Fig. 1F, crosses). As spikes are needed to calculate the impulse response, if, for any neuron in any condition, the mean spike rate over all trials of that condition was less than 5 spikes s^−1^, all trials in that condition for that neuron were discarded from analysis. This resulted in two neurons in the inhibitory roll −25 deg s^−1^ condition and four neurons in the inhibitory roll –50 deg s^−1^ condition being discarded.

**Fig. 3.**
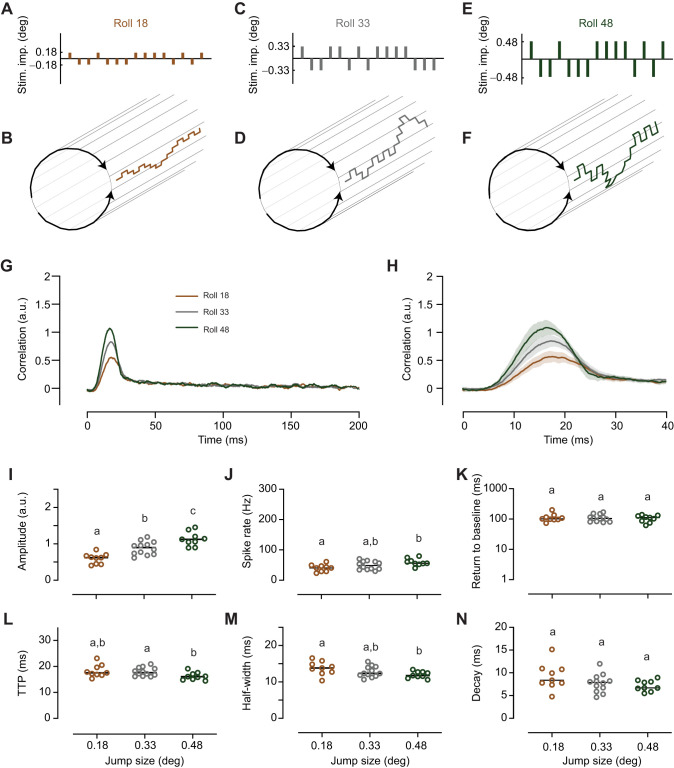
**The roll impulse response scales with the size of the stimulus impulse.** (A) To control roll motion, we used an m-sequence which described a series of 0.18 deg stimulus impulses in the preferred (positive) and anti-preferred (negative) direction (part of the m-sequence shown). (B) When controlling roll motion, this consists of a series of excitatory and inhibitory roll rotation increments over time (note that the rotation increments are not to scale). (C) The Roll 33 stimulus consisted of a series of 0.33 deg stimulus impulses in the preferred (positive) and anti-preferred (negative) direction (part of the m-sequence shown). (D) The Roll 33 m-sequence extract shown in C when projected as excitatory and inhibitory roll rotation increments over time (not to scale). (E) The Roll 48 m sequence consisted of a series of 0.48 deg stimulus impulses (part of the m-sequence shown). (F) The Roll 48 m sequence in E when projected as ER and IR rotation increments over time (not to scale). (G) The mean impulse response to Roll 18 (*N*=9), Roll 33 (*N*=12) and Roll 48 (*N*=9). (H) A magnification of the impulse response (means with 95% confidence intervals). (I) The maximum amplitude of the impulse response across neurons. (J) The mean spike rate as a function of the size of the stimulus impulse. (K) The return to baseline as a function of stimulus impulse size. (L) The TTP of the impulse response. (M) The half-width of the impulse response. (N) The decay time. In I–N, the horizontal lines show the median, and different letters above the data points indicate significant differences from *post hoc* pairwise comparisons (Mann–Whitney *U*), with Benjamini–Hochberg correction, with *P*<0.05.

**Fig. 4.**
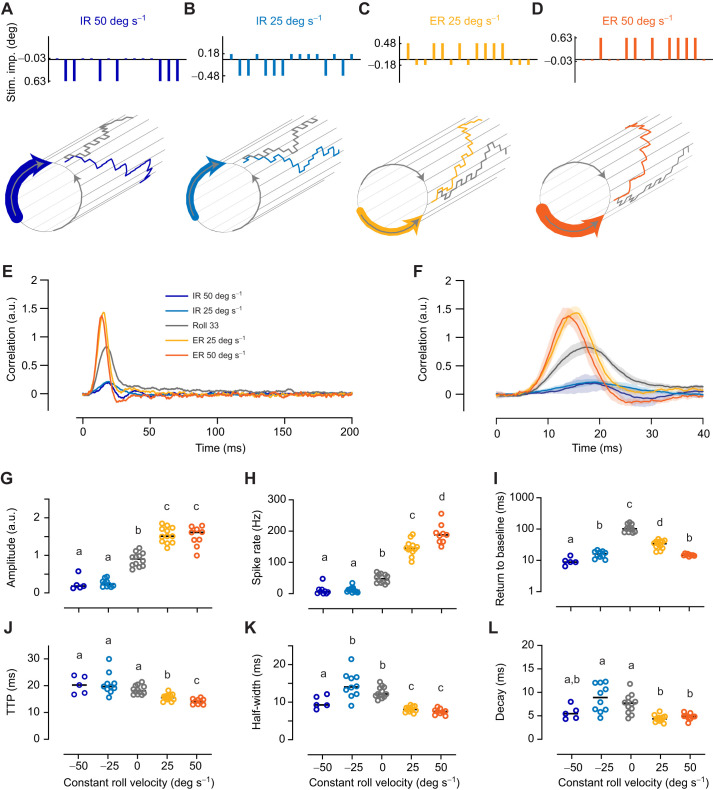
**The roll impulse response is affected by the addition of constant roll.** (A) To control roll motion, we used an m-sequence which described a series of 0.33 deg stimulus impulses in the preferred and anti-preferred direction (gray). We added inhibitory roll (IR) at 50 deg s^−1^ (dark blue arrow), which when displayed on a monitor with 165 Hz temporal resolution is a series of anti-preferred direction rotations of 0.3 deg each. The resulting stimulus thus consisted of preferred direction impulses of 0.03 deg each, and anti-preferred direction impulses of 0.63 deg each (blue). (B) After adding IR at 25 deg s^−1^, our stimulus consisted of preferred direction jumps of 0.18 deg each and anti-preferred direction jumps of 0.48 deg each. (C) The roll m-sequence after adding ER at 25 deg s^−1^. (D) The roll m-sequence after adding ER at 50 deg s^−1^. (E) The mean impulse response to IR at 50 deg s^−1^ (*N*=5), IR at 25 deg s^−1^ (*N*=10), Roll 33 (*N*=12), ER at 25 deg s^−1^ (*N*=12) and ER at 50 deg s^−1^ (*N*=9). (F) A magnification of the impulse responses shown in E (means with 95% confidence intervals). (G) The impulse response amplitude as a function of constant roll velocity from the neurons shown in E and F. (H) The spike rate as a function of constant roll velocity. (I) The return to baseline. (J) The TTP of the impulse response. (K) The half-width of the impulse response. (L) The decay time. The rotation increments on the cylinders in A–D are not to scale. In E–L, the Roll 33 data are replotted from Fig. 3. In G–L, the horizontal lines show the median values, and different letters above the datasets indicate significant differences from *post hoc* pairwise comparisons (Mann–Whitney *U*), with Benjamini–Hochberg correction, with *P*<0.05.

**Fig. 5.**
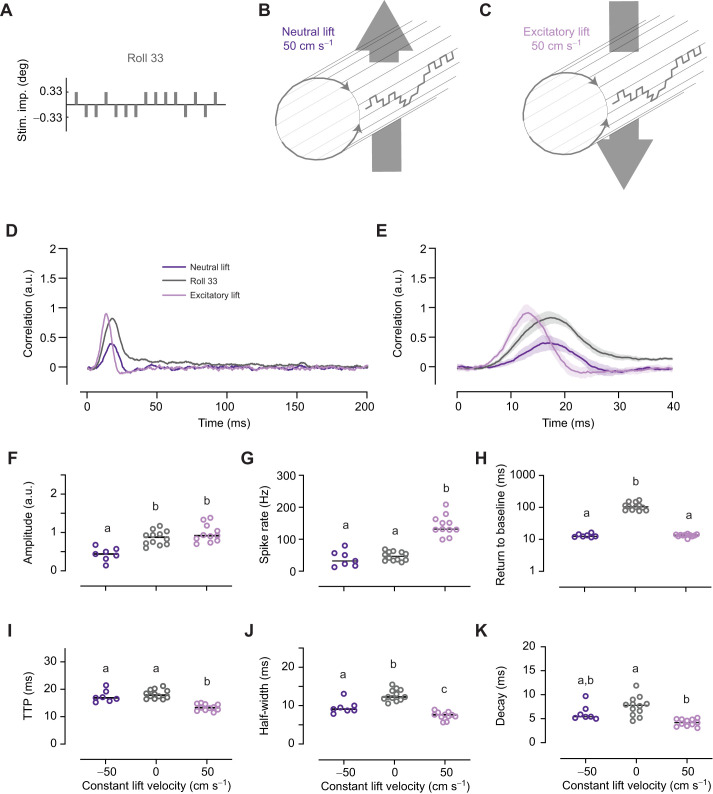
**The impulse response to roll motion is affected by lift translation.** (A) To control roll motion, we used an m-sequence which described a series of 0.33 deg stimulus impulses. The pictogram shows an extract of a Roll 33 m-sequence. (B) We added neutral lift at 50 cm s^−1^, i.e. a series of anti-preferred direction impulses of 0.3 cm each, to the Roll 33 m-sequence. (C) We added excitatory lift at 50 cm s^−1^. (D) The mean impulse response to neutral lift (*N*=7), Roll 33 (*N*=12) and excitatory lift (*N*=11). (E) A magnification of the impulse responses (means with 95% confidence intervals). (F) The maximum amplitude of the impulse response across neurons as a function of constant lift velocity. (G) The spike rate as a function of constant lift velocity. (H) The return to baseline. (I) The TTP as a function of constant lift velocity. (J) The half-width of the roll impulse response. (K) The decay time. In D–K, the Roll 33 data are replotted from Fig. 3. In F–K, the horizontal lines show the median, and different letters above the data points indicate significant differences from *post hoc* pairwise comparisons (Mann–Whitney *U*), with Benjamini–Hochberg correction, with *P*<0.05.

All figures were prepared in Graphpad Prism 9.2.0 (Graphpad Software). The data in Fig. 1F were statistically analyzed using a one-way ANOVA followed by Dunnett's multiple comparison test using Graphpad Prism 9.2.0. The parameters extracted from mean impulse responses shown in Figs 3–5 were first analyzed using an omnibus Kruskal–Wallis test comprising all nine experimental conditions, and using the Pingouin statistical software package (Vallat, 2018). This showed a significant effect of condition for spike rate (*H*=78.298, d.f.=8, *P*<0.001), amplitude (*H*=72.416, d.f.=8, *P*<0.001), TTP (*H*=58.972, d.f.=8, *P*<0.001), half-width (*H*=65.492, d.f.=8, *P*<0.001), decay (*H*=50.574, d.f.=8, *P*<0.001) and return to baseline (*H*=72.123, d.f.=8, *P*<0.001). We then used the Mann–Whitney *U* test for pairwise comparisons and corrected for multiple comparisons using the Benjamini–Hochberg method. A significance threshold of *P*<0.05 was used throughout.

Percentage change was defined as (Median_experimental condition_−Median_Roll33_)/Median_Roll33_.

Raw and analyzed data, as well as required analysis scripts are available from Dryad (https://doi.org/10.5061/dryad.pzgmsbcm0).

## RESULTS

### Optic flow sensitivity

To investigate impulse responses of optic flow-sensitive descending neurons in male *E. tenax*, we used a starfield stimulus that simulated a 3-dimensional space around the hoverfly (Nicholas et al., 2020). For this purpose, we simulated a cube with 4 m sides, with the hoverfly placed in its center (Fig. 1A, not to scale), containing 2 cm diameter spheres at a density of 100/m^3^. We projected the ca. 1200 spheres that were located in the anterior visual field onto a 2-dimensional screen placed in front of the hoverfly, with size and grayscale used to indicate distance (Fig. 1B, not to scale). For example, in Fig. 1A, there are three spheres in the part of the anterior visual field encompassing the screen, located at different distances from the hoverfly. When projected onto the 2-dimensional screen, the sphere that is closest (Fig. 1B, black, upper left) is rendered larger than the sphere that is furthest away (Fig. 1B, light gray, bottom right).

We recorded extracellularly from optic flow-sensitive descending neurons. We confirmed that we were recording from type 2 neurons by quantifying the response to optic flow (Fig. 1C–F) and widefield sinusoidal gratings, and by mapping the receptive field of each neuron (Nicholas et al., 2020). To quantify the response to roll and lift, we displayed constant velocity optic flow for 1 s and calculated the mean spike rate for the entire peristimulus duration (Fig. 1D,E, shaded area), bar the first 100 ms (Fig. 1D,E, boxed area). The response was then compared with the spike rate during the 500 ms immediately preceding stimulation (Fig. 1F, spontaneous). As previously (Nicholas et al., 2020), we found that type 2 neurons that have their receptive fields in the right visual field (*N*=5) were strongly excited by roll moving clockwise on the screen, as seen by the hoverfly, whereas counter-clockwise roll excited those with receptive fields in the left visual field (*N*=8). The responses from neurons with receptive fields in the right visual field were assumed to be mirror images of those in the left visual field, and from here on we refer to counter-clockwise roll on the screen as excitatory, and clockwise roll as inhibitory. As previously (Nicholas et al., 2020), we found that lift optic flow moving downward as seen by the hoverfly excited the type 2 descending neurons, whereas upwards lift gave no response above spontaneous rate (Fig. 1F, one-way ANOVA followed by Dunnett's multiple comparison test, *P*=0.17), and is therefore referred to as ‘neutral lift’ from here on. We excluded one neuron that gave a low response to preferred direction roll (Fig. 1F, crosses).

### Impulse response to roll optic flow

We next determined the impulse response to roll motion, by presenting a randomly selected m-sequence (Aptekar et al., 2014). Each m-sequence was presented at 165 Hz, with each stimulus impulse rotating the starfield pattern by 0.33 deg (extract example shown in Fig. 2A), which would correspond to a velocity of 50 deg s^−1^ if all stimulus impulses moved in the same direction. We refer to this stimulus as Roll 33. Fig. 2B shows an example raw data trace recorded from a type 2 descending neuron in response to the stimulus shown in Fig. 2A (see also Movie 1), and Fig. 2C shows the resulting spike train after spike sorting the raw data. The m-sequence consisted of a series of positive and negative increments (Fig. 2A). As these were used to control roll rotation, this resulted in a series of 0.33 deg stimulus impulses in the counter-clockwise (excitatory roll, ER; Fig. 2D,E, not to scale) and clockwise direction (inhibitory roll, IR; Fig. 2D,E, not to scale), as seen by the hoverfly on the screen.

The impulse response to roll (Fig. 2F) was calculated by cross-correlation of the m-sequence controlling the stimulus (Fig. 2A) with the neural response (Fig. 2C). From each neuron's average impulse response, we quantified its peak amplitude, TTP and half-width (Fig. 2F,G), as these are commonly used to quantify impulse responses (e.g. Behnia et al., 2014; Fox et al., 2014; Lee et al., 2015; Osorio, 1991). In addition, we noted that it took a variable time for the impulse response to return to baseline levels. To capture this observation, we quantified the return to baseline, defined as the time between the TTP and a return of the smoothed firing rate to within 2 standard deviations of the baseline (Fig. 2F, blue). We also quantified the decay time, defined as the time it took to return to 1/*e* of maximum amplitude, which can be seen as an estimate of an exponential decay (Fig. 2G).

Fig. 2H shows the impulse response to Roll 33 across neurons. As a validation of this impulse response, we additionally shuffled all spike trains before doing the cross-correlation. The impulse responses calculated using such randomized spike trains were flat (Fig. 2I), suggesting that the Roll 33 impulse response (Fig. 2H) is not an artefact of our analysis.

We additionally validated the m-sequence method by predicting the response to a shared Roll 33 m-sequence, seen by all neurons. For each neuron, we first calculated the mean Roll 33 impulse response, using all repetitions except the one featuring the shared m-sequence. In addition, we added a non-linearity to the response (see Materials and Methods) to predict the spiking response. We then convolved the mean impulse response with the held-out shared m-sequence and passed the result through the non-linearity, in order to predict the response to the held-out m-sequence. This prediction was compared with the mean recorded response to the held-out m-sequence across the other, held-out neurons. We found that the median correlation between the predicted and recorded response (Fig. 2J, *N*=12) was 0.725 (range: 0.552–0.763).

For comparison, we repeated the above process 20 times for each neuron, each time using randomly shuffled spike data (shuffling was carried out as described above), to derive a ‘baseline’ impulse response and non-linearity. The median correlation of the prediction based on shuffled data with the recorded response was 0.000 (range: −0.146–0.081).

### Impulse response depends on stimulus size

To investigate whether the roll impulse response depends on the size of the stimulus impulse, we used three different impulse sizes: 0.18 deg (Roll 18; Fig. 3A,B), 0.33 deg (Roll 33; Fig. 3C,D; Movie 1) and 0.48 deg (Roll 48; Fig. 3E,F). If the stimulus impulses had all been in the same direction, when shown at 165 Hz, they would correspond to velocities of 30, 50 and 80 deg s^−1^, respectively. We found that the roll impulse response amplitude increased with the size of the stimulus impulse (Fig. 3G–I). Indeed, when the stimulus impulse size was 0.18 deg instead of 0.33 deg, the median amplitude decreased by 31%, and when the stimulus impulse size increased from 0.33 deg to 0.48 deg, the median amplitude increased by 26%. In addition, we found that the spike rate during the presentation of the m-sequence scaled linearly with the size of the stimulus impulse, but the effect was only significant between Roll 18 and Roll 48 (Fig. 3J; Fig. S1A).

We next looked at the timing of the impulse response and found that the median return to baseline was 99.7–109.1 ms (Fig. 3K). However, there was no significant effect of stimulus impulse size (Fig. 3K; Fig. S1B). The TTP of the impulse response decreased slightly with stimulus impulse size, with a median TTP of 17.9 ms for Roll 18, 18.0 ms for Roll 33 and 16.5 ms for Roll 48, but the effect was only significant between Roll 33 and Roll 48 (Fig. 3L; Fig. S1C). The half-width also decreased with stimulus impulse size, with a median half-width of 13.8 ms for Roll 18, 12.3 ms for Roll 33 and 11.7 ms for Roll 48, but the effect was only significant between Roll 18 and Roll 48 (Fig. 3M; Fig. S1D). The TTP and half-width are thus similar to what has previously been measured in LPTCs in hoverflies (Lee et al., 2015) and *Drosophila* (Schnell et al., 2017). However, note that the LPTC impulse responses were recorded using other types of widefield stimuli. We also found that the size of the stimulus impulse did not have a significant effect on the impulse response decay (Fig. 3N; Fig. S1E).

### Addition of ER makes the roll impulse response faster and stronger

In blowflies, the yaw spike-triggered average in H1 neurons is affected by the addition of either roll or pitch (Roy et al., 2015). To investigate whether the roll impulse response in optic flow-sensitive descending neurons is affected by constant optic flow, we added inihibitory or excitatory roll at 25 or 50 deg s^−1^ to Roll 33 m-sequences (Movies 2 and 3). Roll optic flow is important for hoverflies for performing stabilizing head reflexes during body rotations (Goulard et al., 2015). When roll rotating at 50 deg s^−1^ (Fig. 4A, blue arrow) is displayed on a visual display with 165 Hz temporal resolution, it results in a series of 0.3 deg rotation increments. When this is added to the underlying randomly selected m-sequence, which is also run at 165 Hz (Fig. 4A, gray), the resulting stimulus consists of impulses that are either 0.03 deg in the excitatory direction or 0.63 deg in the inhibitory direction (Fig. 4A, blue; Movie 2). Similarly, adding inhibitory roll at 25 deg s^−1^ results in 0.15 deg increments when displayed at 165 Hz. The resulting stimulus therefore consists of impulses that are either 0.18 deg in the inhibitory direction or 0.48 deg in the excitatory direction (Fig. 4B, blue). We also added excitatory roll, using the same logic (Fig. 4C,D; Movie 3).

We found that the Roll 33 impulse response was strongly influenced by the addition of excitatory or inhibitory roll (Fig. 4E,F, compare gray and colored data). Indeed, the median impulse response amplitude decreased by 81% with the addition of IR at 50 deg s^−1^, and by 75% with the addition of IR at 25 deg s^−1^ (Fig. 4E–G). Conversely, the median impulse response amplitude increased by 68% with the addition of ER at 25 deg s^−1^, and by 80% with the addition of ER at 50 deg s^−1^ (Fig. 4E–G). We found that the spike rate was significantly affected by the addition of inhibitory as well as ER (Fig. 4H; Fig. S1A).

The long return to baseline of the Roll 33 impulse response (Fig. 4E,I, gray) was significantly reduced with the addition of either ER or IR (Fig. 4E,I, colored; Fig. S1B). The Roll 33 TTP (Fig. 4J; Fig. S1C) and decay (Fig. 4L; Fig. S1E) were not strongly affected by the addition of IR. The Roll 33 impulse response half-width decreased by 24% with the addition of IR at 50 deg s^−1^, but there was no significant effect at 25 deg s^−1^ (Fig. 4K; Fig. S1D). In contrast, TTP, half-width and decay all decreased with the addition of ER. Indeed, the median TTP decreased by 22%, the half-width by 38% and the decay by 36% when ER at 50 deg s^−1^ was added to the Roll 33 m-sequence (Fig. 4J–L, red). In summary, IR gave the Roll 33 impulse responses a lower amplitude (Fig. 4, blue), whereas excitatory roll made the impulse response larger, faster and narrower (Fig. 4, yellow and red).

### Lift optic flow affects the roll impulse response

To investigate whether the roll impulse response in descending neurons is affected by other types of constant optic flow, we added neutral or excitatory lift at 50 cm s^−1^ to the Roll 33 m-sequence (Fig. 5A–C; Movies 4 and 5). *Drosophila* and blowfly DNOVS2, as well as the hoverfly type 2 neuron, respond strongly not just to roll motion but also to lift optic flow (Haag et al., 2007; Nicholas et al., 2020; Suver et al., 2016). Continuous lift motion would be experienced if a fly is, for example, free falling. We found that adding neutral lift to the roll m-sequence decreased the median impulse response amplitude by 49% (Fig. 5D–F, compare gray and dark purple) even if the median spike rate was unaffected (Fig. 5G, dark purple; Fig. S1A). In contrast, we found that adding excitatory lift to the roll m-sequence did not affect the impulse response amplitude (Fig. 5D–F, compare light purple and gray), even if the median spike rate increased by 178% (Fig. 5G, light purple; Fig. S1A). We found that the slow return to baseline of the Roll 33 impulse response disappeared with the addition of lift in either direction (Fig. 5D,H, compare gray and purple; Fig. S1B).

The TTP of the Roll 33 impulse response was unaffected by neutral lift, but decreased by 25% with the addition of excitatory lift (Fig. 5D,E,I; Fig. S1C). The half-width of the Roll 33 impulse response decreased with the addition of neutral lift (by 26%, Fig. 5D,E,J) as well as excitatory lift (by 38%, Fig. 5D,E,J; Fig. S1D). The decay also decreased with the addition of excitatory lift (by 46%, Fig. 5D,E,K). None of these effects could be explained solely on the basis of the changed spike rate (Fig. S1B–E).

## DISCUSSION

We found that the TTP and half-width of the roll impulse response (Fig. 3G,H,L,M) was similar to what has been reported for impulse responses to a range of different widefield stimuli in a range of different LPTCs, including *Eristalis* HS cells (Lee et al., 2015), *Drosophila* HS cells (Schnell et al., 2014) and blowfly H1 (Roy et al., 2015). For example, *Eristalis* HS cell impulse responses to widefield horizontal motion have a TTP of 18.3 ms and a half-width of 10 ms (Lee et al., 2015), and we found here that the type 2 descending neuron Roll 33 TTP was 18.0 ms (Fig. 3L) and its half-width was 12.3 ms (Fig. 3M). The similar time course is interesting because the LPTCs and descending neurons are probably linked via both chemical and electrical synapses (Haag et al., 2007). For example, dual recordings between VS cells and DNOVS1 in blowflies have shown that there is a negligible delay between them. Indeed, injecting a current in either neuron shows a cross-correlation which peaks at 0 ms, with a half width of 3.2 ms (Haag et al., 2007), as expected for electrical coupling. This could thus explain the similar TTP values that we recorded in optic flow-sensitive descending neurons compared with LPTCs.

It is thus not surprising to see such fast impulse responses to roll m-sequences (TTP, Figs 3–5). However, impulse responses recorded in behavior are much more sluggish (e.g. Aptekar et al., 2012). Indeed, *Drosophila* work measuring impulse responses in both HS cells and behavior suggested that this difference could be caused by accumulating calcium at the output synapse of the LPTCs (Schnell et al., 2014), acting as a leaky integrator. Indeed, our own work showed that the descending neurons displayed some adaptation effects typical of LPTCs, but also persistent firing following preferred-direction stimulation, potentially generated by this accumulating calcium (Nicholas and Nordström, 2020). Such accumulating calcium and its resulting persistent firing could possibly explain the slow return to baseline that we saw in our recordings (e.g. Fig. 3K). Indeed, the median return to baseline of the Roll 33 impulse response was 102 ms. Interestingly, though, adding constant roll (Fig. 4I) or lift (Fig. 5H) in either direction substantially and significantly decreased the return to baseline, and we found no correlation with the spike rate of the neuron (Fig. S1B). Maybe this is because the descending neurons collate information from many different LPTCs (Haag et al., 2007; Suver et al., 2016), and when using different types of optic flow, the input from these is optimally encoded by different LPTCs, and this affects the sustained response component. Indeed, considering that the optic lobe harbors 45–60 LPTCs (Pierantoni, 1976), whereas there are only a handful of optic flow-sensitive descending neurons (Namiki et al., 2018; Suver et al., 2016), there must be substantial neural pooling taking place. The long return to baseline that we recorded is nevertheless far from the finding for behavioral impulse responses where, for example, the roll impulse response does not return to baseline for over 1 s (e.g. Aptekar et al., 2012; Theobald et al., 2010a), and even if the thrust impulse response decays back to baseline much more quickly (Theobald et al., 2010a), the yaw impulse response takes even longer (Fox et al., 2014; Schnell et al., 2014).

These relatively sluggish behavioral return-to-baseline observations, together with much slower TTP (Schnell et al., 2014), are seen despite the fact that flight responses can be extremely rapid, with delays as short as 10 ms recorded from the blowfly *Lucilia* (Varennes et al., 2020). Indeed, the impulse response of *Drosophila* behavior peaks after several hundred milliseconds, whereas we saw a Roll 33 TTP of 18.0 ms (Fig. 3L). However, a precaution to take when interpreting these results is that visual information can be gated. For example, in the neck motor neurons, visual information is gated by haltere motion (Huston and Krapp, 2009), suggesting that the descending neurons might behave differently in flying animals than in immobilized preparations, such as here. Indeed, physical activity is known to shift the motion sensitivity of LPTCs (Longden and Krapp, 2009; Maimon et al., 2010), and could probably have a big effect on the descending neurons too. In future work it would therefore be important to record behavioral and neurophysiological impulse responses at different stages of the entire sensorimotor transformation cascade for more direct comparisons (e.g. Schnell et al., 2014), and to quantify the role internal state such as arousal may have (Rosner et al., 2010). Furthermore, it would be beneficial to compare neural and behavioral impulse responses in closed-loop settings as efference copies have a strong influence on the processing of widefield motion (Fenk et al., 2021).

We added constant roll or lift optic flow to some of our stimuli (Figs 4 and 5). Roll optic flow is important for head-stabilizing reflexes, and at least in experimental settings, hoverflies can continue to perform head rotations to stabilize the horizon for several seconds (Goulard et al., 2015). Lift optic flow is important for being able to correct for free-falling conditions (Goulard et al., 2018). However, roll or lift optic flow continuing for several seconds would probably not occur often in natural conditions. Nevertheless, we found that the addition of constant roll or lift affected the roll impulse response of optic flow-sensitive descending neurons (Figs 4 and 5). For example, excitatory roll made the roll impulse responses faster (Fig. 4E,F,J), larger (Fig. 4E–G) and narrower (Fig. 4E,F,K,L). Excitatory lift also made the roll impulse responses faster (Fig. 5D,E,I) and narrower (Fig. 5D,E,J,K), but not larger (Fig. 5D–F). This is despite the increased spike rate suggesting that the excitation level was similar (compare Fig. 4H, yellow and red with Fig. 5G, light purple). Indeed, when increasing the size of the stimulus impulse, both the spike rate (Fig. 3J) and the impulse response amplitude increased (Fig. 3G–I), suggesting that these are correlated under some conditions (see also Fig. S1A).

In previous work using white noise stimuli in blowfly H1 neurons, it was found that the addition of either excitatory roll or inhibitory pitch decreased the decay time constant of the yaw spike-triggered average (Roy et al., 2015). This is consistent with some features that we saw in our roll impulse responses. For example, we saw that both the decay and the return to baseline of the roll impulse response decreased with the addition of constant roll or lift (Figs 4I,L and 5H,K). In addition, the decreased impulse response amplitude with the addition of inhibitory stimuli (Figs 4G and 5F) is consistent with observations in blowfly H1 (Roy et al., 2015). However, other effects are not consistent with previous H1 results. For example, we found that the TTP decreased with the addition of excitatory stimuli (Figs 4J and 5I), but in H1 additional excitatory stimuli did not affect the TTP of the spike-triggered average (Roy et al., 2015). In *Drosophila* behavior, however, the sideslip impulse response became faster and narrower with the addition of constant sideslip, but not with constant thrust (Theobald et al., 2010b). The descending neurons did not show a corresponding difference depending on whether we added constant roll (Fig. 4) or constant lift (Fig. 5).

We found that the addition of inhibitory roll gave the roll impulse response a smaller amplitude (Fig. 4E–G) and half-width (Fig. 4E,F,K), similar to what happened with the addition of neutral lift (Fig. 5D–F,J). It is important to note that in a spiking neuron we can only quantify the impulse response when there are spikes. Therefore, this could have skewed the quantification of the impulse response towards neurons that were more likely to respond. Indeed, we could not extract an impulse response in two neurons after adding IR at 25 deg s^−1^ and four neurons after adding IR at 50 deg s^−1^. However, note that neutral lift affected the impulse response similarly to IR (compare Fig. 4, blue data with Fig. 5, dark purple data). This is interesting as neutral lift did not inhibit the neurons’ spike rate (Figs 1D and 5G). This therefore suggests that neutral lift is inhibitory, but only if the neuron is already excited. More work investigating the interactions between different types of optic flow is clearly needed.

## Supplementary Material

Supplementary information
