# Supplementary material for: The impulse response of optic flow-sensitive descending neurons to roll m-sequences
Source: J Exp Biol. 2021 Dec 6;224(23):jeb242833. doi: 10.1242/jeb.242833 (PMC8714074; doi:10.1242/jeb.242833)
Supplement: Supplementary information [file jexbio-224-242833-s1.pdf]

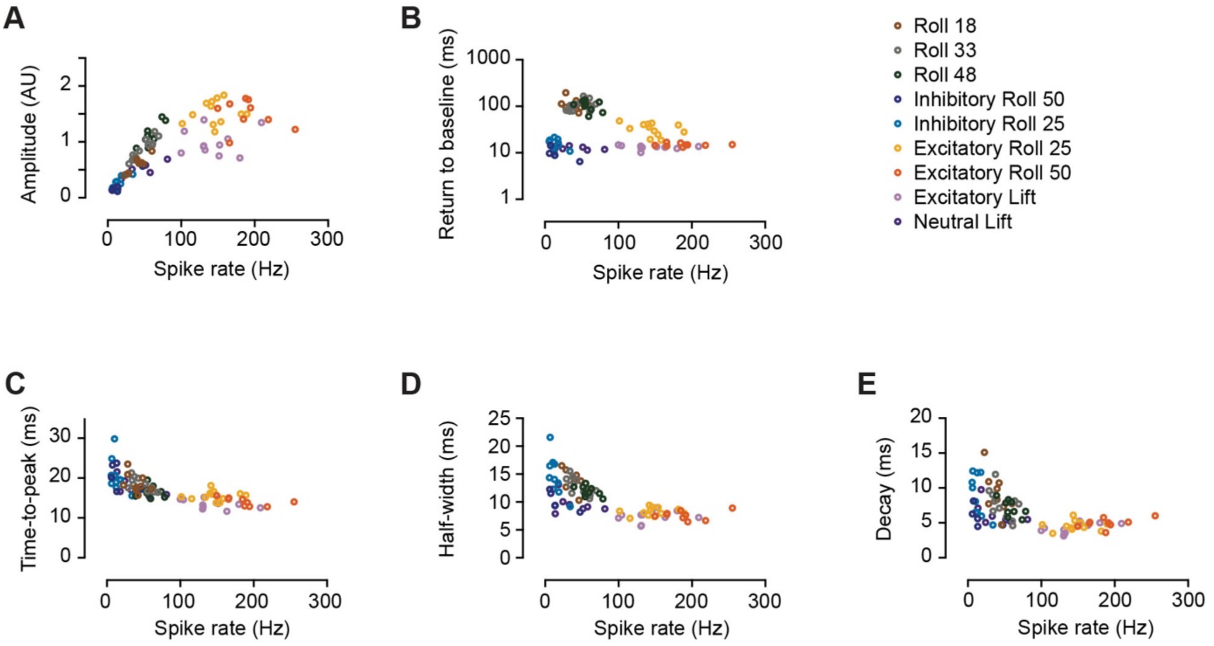

Fig. S1. The spike rate does not explain the effect constant velocity have on the roll impulse response

A) The roll impulse response amplitude of each neuron and each condition as a function of spike rate, color coded according to the stimulus (see Figs. 3-5 of the main paper for full explanation of each stimulus). B) The return to baseline of the impulse response as a function of spike rate. C) The time-to-peak of the impulse response as a function of spike rate. D) The half-width of the impulse response as a function of spike rate. E) The decay of the impulse response as a function of spike rate.

Journal of Experimental Biology • Supplementary information

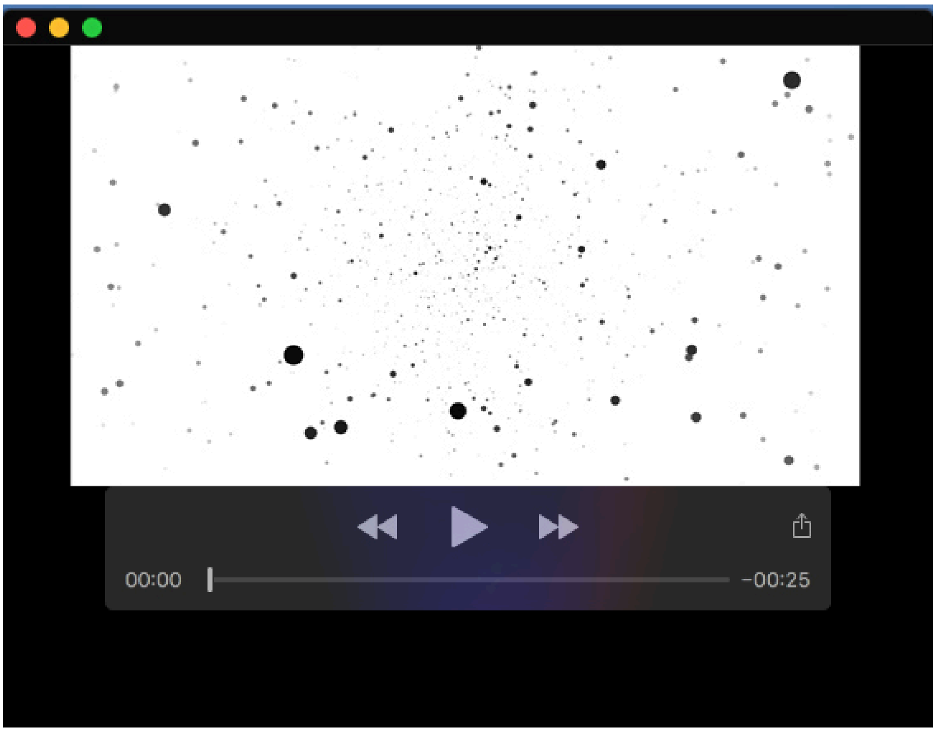

Movie 1. Roll 33 stimulus

The movie shows an example of a Roll 33 m-sequence. The hoverfly would have watched this stimulus at a distance of 6.5 cm, placed perpendicular to the screen, with its center of gaze oriented towards the center of the screen. At this distance the visual stimulus subtended 155 degrees azimuth x 138 degrees elevation.

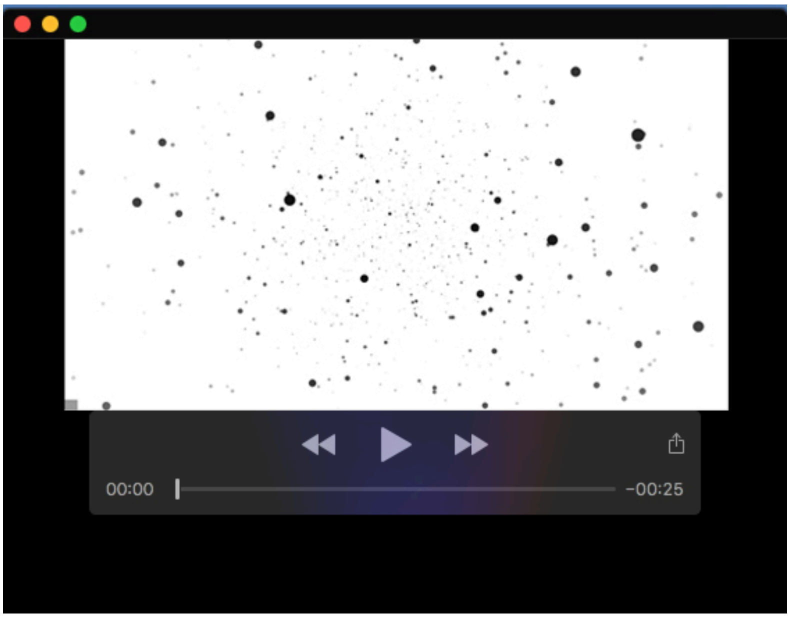

Movie 2. Inhibitory Roll 50 stimulus

The movie shows an example of an Inhibitory Roll 50 m-sequence.

Journal of Experimental Biology • Supplementary information

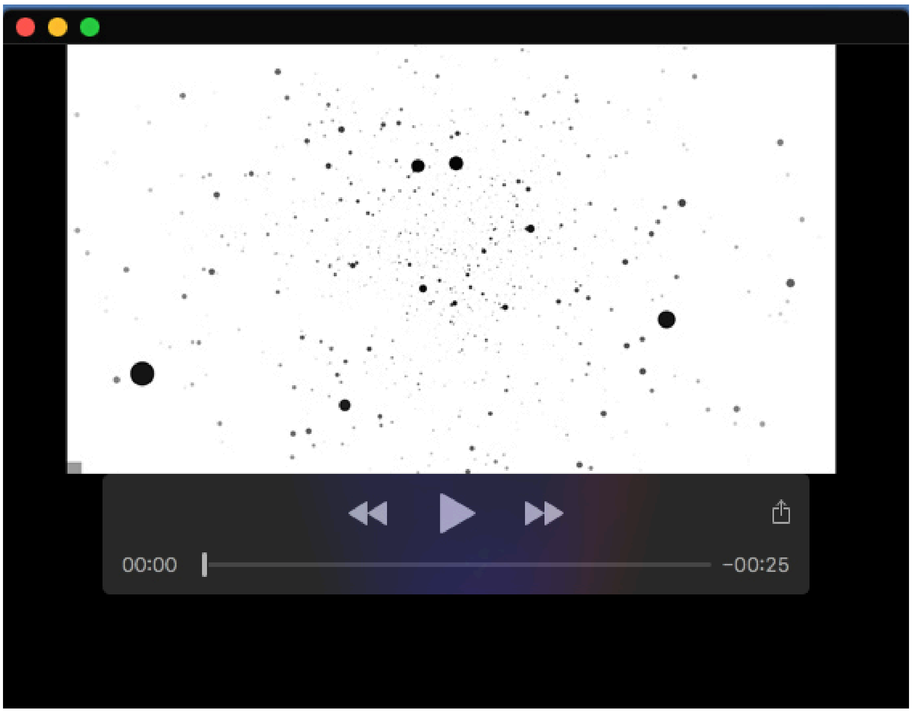

Movie 3. Excitatory Roll 50 stimulus

The movie shows an example of an Excitatory Roll 50 m-sequence.

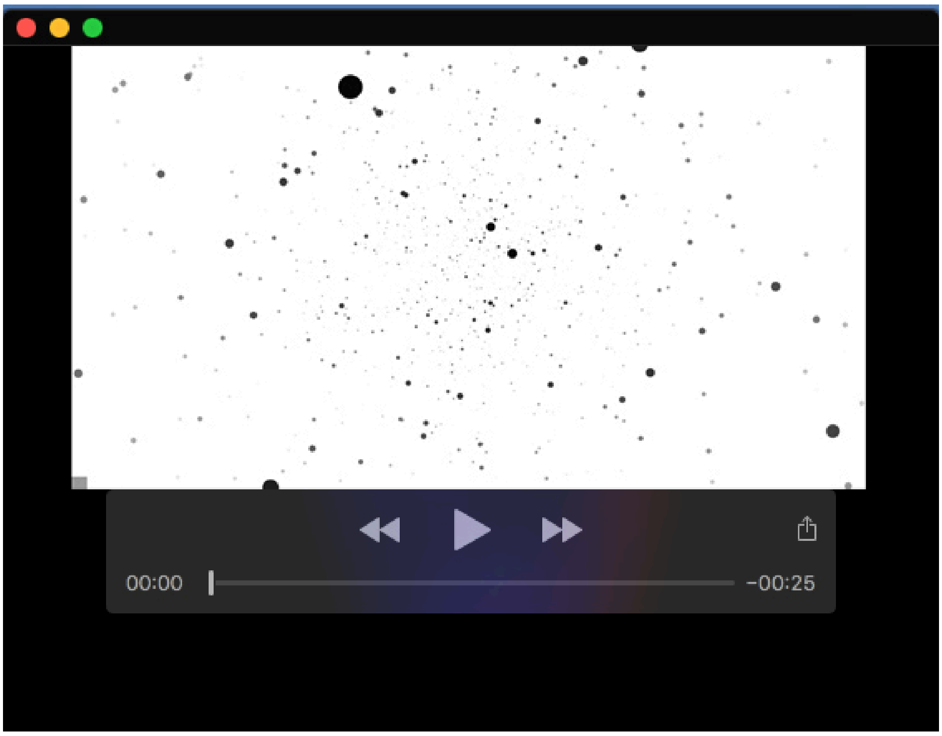

Movie 4. Neutral Lift stimulus

The movie shows an example of a neutral lift stimulus.

Journal of Experimental Biology • Supplementary information

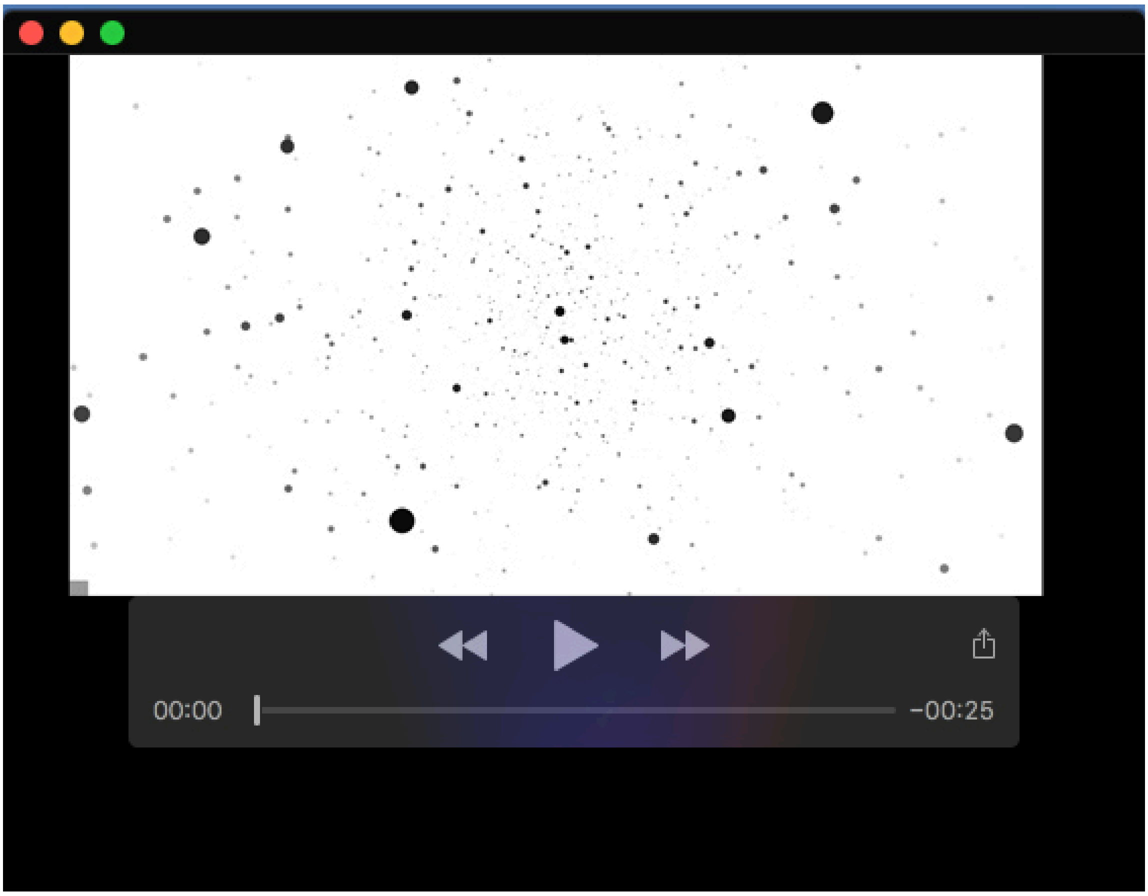

Movie 5. Excitatory Lift stimulus

The movie shows an example of the excitatory lift stimulus.
